# Non-invasive Real-Time Hemodynamic Monitoring During Transcatheter Aortic Valve Replacement: A Case Series

**DOI:** 10.7759/cureus.83468

**Published:** 2025-05-04

**Authors:** Zain Khalpey, Usman Aslam, Tyler Phillips, Elliott Davis, Zacharya I Khalpey, Ujjawal Kumar, Feras H Khaliel

**Affiliations:** 1 Department of Cardiac Surgery, HonorHealth, Scottsdale, USA; 2 Khalpey AI Lab, Applied & Translational AI Research Institute (ATARI), Scottsdale, USA; 3 Department of General Surgery, HonorHealth, Phoenix, USA; 4 Department of Cardiothoracic Surgery, HonorHealth, Scottsdale, USA; 5 School of Clinical Medicine, University of Cambridge, Cambridge, GBR; 6 Department of Cardiac Surgery, King Faisal Specialist Hospital and Research Centre, Riyadh, SAU

**Keywords:** blood pressure, hemodynamic monitoring, noninvasive blood pressure monitoring, transcatheter aortic valve replacement, vitalstream

## Abstract

This study analyzes arterial pressure rise times during transcatheter aortic valve replacement (TAVR) procedures across four cases. We demonstrate that successful TAVR deployment is associated with immediate improvements in arterial pressure wave characteristics, specifically showing decreased rise times, suggesting enhanced hemodynamic performance post-valve deployment. We also demonstrate a correlation between established methods of hemodynamic monitoring (arterial line, blood pressure cuff) and a novel non-invasive device.

## Introduction

Accurate hemodynamic management and assessment are crucial in cardiac surgical care [1]. Real-time assessment goes beyond traditional monitoring standards, monitoring oxygenation, ventilation, circulation, and temperature. Cardiac surgical patients also require monitoring of blood pressure, blood flow, intravascular volume, vascular tone, and cardiac function. Invasive monitoring methods like pulmonary artery catheters may not be beneficial in low-risk patients undergoing low-risk cardiac surgery [2]. Advancements in non-invasive monitoring offer alternative tools to assess hemodynamics and guide resuscitation. The VitalStream (VS) continuous physiological monitor (Caretaker Medical LLC, Charlottesville, VA) uses distal pulse measurements to perform pulse decomposition analysis and measure advanced hemodynamic parameters. It has been studied in post-cardiac surgery intensive care unit patients and shows agreement with continuous, invasive methods for cardiac output estimation [3]. Similarly, it may be used intra-operatively to measure comparable parameters like the PAC.

Transcatheter aortic valve replacement (TAVR) is a minimally invasive procedure to treat severe aortic stenosis. It is particularly used in patients considered high-risk for conventional surgical aortic valve replacement (SAVR) [4,5]. Aortic stenosis, characterized by a narrowed aortic valve opening, obstructs blood flow and increases cardiac workload, potentially leading to heart failure if untreated [6]. TAVR offers a safer alternative to SAVR, which involves open-heart surgery with risks and extended recovery times. It involves the percutaneous insertion of a bioprosthetic valve via a catheter, typically through the femoral artery, though other routes such as transcarotid or transapical access are also performed. TAVR has reduced procedural morbidity and mortality, shortened hospital stays, and improved recovery times compared to SAVR [7]. Since its approval, TAVR has expanded to include intermediate and even low-risk patients [8,9].

Despite literature on TAVR’s long-term outcomes, there is limited data on the immediate hemodynamic changes during the procedure [6]. Understanding these changes is crucial for real-time feedback on valve deployment and postoperative function. Arterial pressure risetime, reflecting the rate of pressure increase during systole, can indicate improved cardiac output and valve function post-deployment.

Invasive monitoring methods like arterial lines provide accurate data but carry risks like infection, bleeding, and vascular complications [10]. Non-invasive techniques could mitigate these risks and simplify the workflow. The VS device, a non-invasive alternative without arterial lines, could simplify the procedure. This series aims to analyze arterial pressure risetimes during TAVR using non-invasive monitoring. The system and the underlying approach have been described in detail elsewhere [11,12]. The device uses a low-pressure (30-40 mmHg), pump-inflated, finger cuff that pneumatically couples arterial pulsations via a pressure line to a pressure sensor for detection and analysis. Data are communicated wirelessly to a tablet-based user interface via Bluetooth or Wi-Fi.

VS tracks aortic blood pressure via pulse decomposition analysis (PDA) of the peripheral pulse at a distal site, typically the finger. The PDA approach is based on the concept that two central reflection sites are primarily responsible for the shape of the pressure pulse envelope of the upper body [13-15]. These two sites (juncture of thoracic and abdominal aortas and the iliac bifurcation) reflect the primary left ventricular ejection pulse to give rise to two component pulses that trail the primary ejection pulse. Within each cardiac cycle, these three component pulses therefore arrive sequentially in the arterial periphery. The spatiotemporal behavior of these three component pulses can be used to monitor hemodynamic states and changes.

We hypothesize that successful TAVR deployment will improve arterial pressure wave characteristics, specifically decreasing risetimes and enhancing hemodynamic performance. Analyzing four cases, we aim to establish a consistent pattern of hemodynamic improvement and explore pulse risetime analysis as a real-time monitoring tool. This paper assesses the measurement of key physiological parameters to evaluate the VS device as a non-invasive alternative for intraoperative monitoring in TAVR. It also presents evidence for quantifying an immediate improvement in arterial pressure wave characteristics, specifically decreased front-end pulse risetimes, suggesting enhanced hemodynamic performance. The VS approach’s passive nature allows for this measurement, unlike active, invasive monitoring systems. We compare arterial catheter- and VS-derived mean arterial pressure (MAP) and heart rate (HRT) measurements, as well as changes in pulse risetimes measured with the VS device before and after TAVR deployment.

## Case presentation

In this case series, we present data for four patients undergoing TAVR at our institution between March 2024 to June 2024. IRB approval requirement was granted for retrospective outcome analysis (23-0025, granted 26th April 2023). All patients provided written informed consent for the TAVR procedures as well as for the use of the VS device.

Clinical characteristics

Table 1 shows the preoperative clinical characteristics of the four patients in this case series. All patients were deemed to be too high risk for surgical aortic valve replacement for their severe calcified aortic stenosis; hence, they underwent TAVR.

**Table 1:** Preoperative characteristics of the four patients BMI: Body mass index; STS: Society of Thoracic Surgeons; CHA2DS2-VASc: Risk score for thrombosis; HAS-BLED: Risk score for major bleeding; OSA: Obstructive sleep apnea; HTN: Hypertension; AFib: Atrial fibrillation; COPD: Chronic obstructive pulmonary disease; MI: Myocardial infarction; PCI: Percutaneous coronary intervention; CKD: Chronic kidney disease.

Variable	Patient 1	Patient 2	Patient 3	Patient 4
Sex	Male	Male	Male	Female
Age (y)	75	83	78	81
BMI (kg/m^2^)	27	27	23	26
STS Score	1.59	4.06	4.24	8.75
CHA2DS2-VASc	4	3	3	5
HAS-BLED	2	2	2	2
Obese	No	No	No	No
OSA	No	No	No	Yes
HTN	Yes	Yes	No	Yes
Prior AFib	Yes	No	Yes	No
Heart Failure	No	No	Yes	No
Hyperlipidemia	Yes	Yes	No	Yes
Diabetes Mellitus	Yes	No	No	Yes
COPD	Yes	No	No	No
Smoking History	Yes	Yes	No	Yes
CAD	No	No	Yes	Yes
Prior MI	No	No	No	No
Prior PCI	No	No	No	No
Prior Cardiac Surgery	No	No	Yes	No
Prior Stroke	Yes	No	No	No
CKD	Yes	No	No	Yes
Immunosuppressive Medications	No	No	No	No

Data collection

Arterial catheter vital sign readings were obtained from the electronic medical record (EMR). The arterial pressure pulse signal was also continuously measured noninvasively using the VS device. In order not to interfere with the surgical procedures, the device was placed on the patient’s wrist during the procedure setup, with the finger cuff coupled to the middle phalanx of the middle finger, and data transmission was verified, after which time no further physical interaction with the device was possible. The device was programmed to perform self-calibration scans at five-minute intervals, operating in between in the continuous tracking mode with the finger cuff pressure collecting pulse data. Collected data were sent via a Wi-Fi interface to an Android tablet for storage. Two distinct risetime measurements were analyzed: full front risetime (10% to 90% of peak amplitude) and partial front risetime (onset to maximum slope point). Outliers and motion artifacts were removed, and the data were smoothed with a 30-point median filter. Procedural events were marked to determine time windows before and after TAVR deployment. Mean values and standard deviations were calculated, and temporal categorizations and pattern consistency were established.

Correlations with invasive monitoring

**Figure 1:**
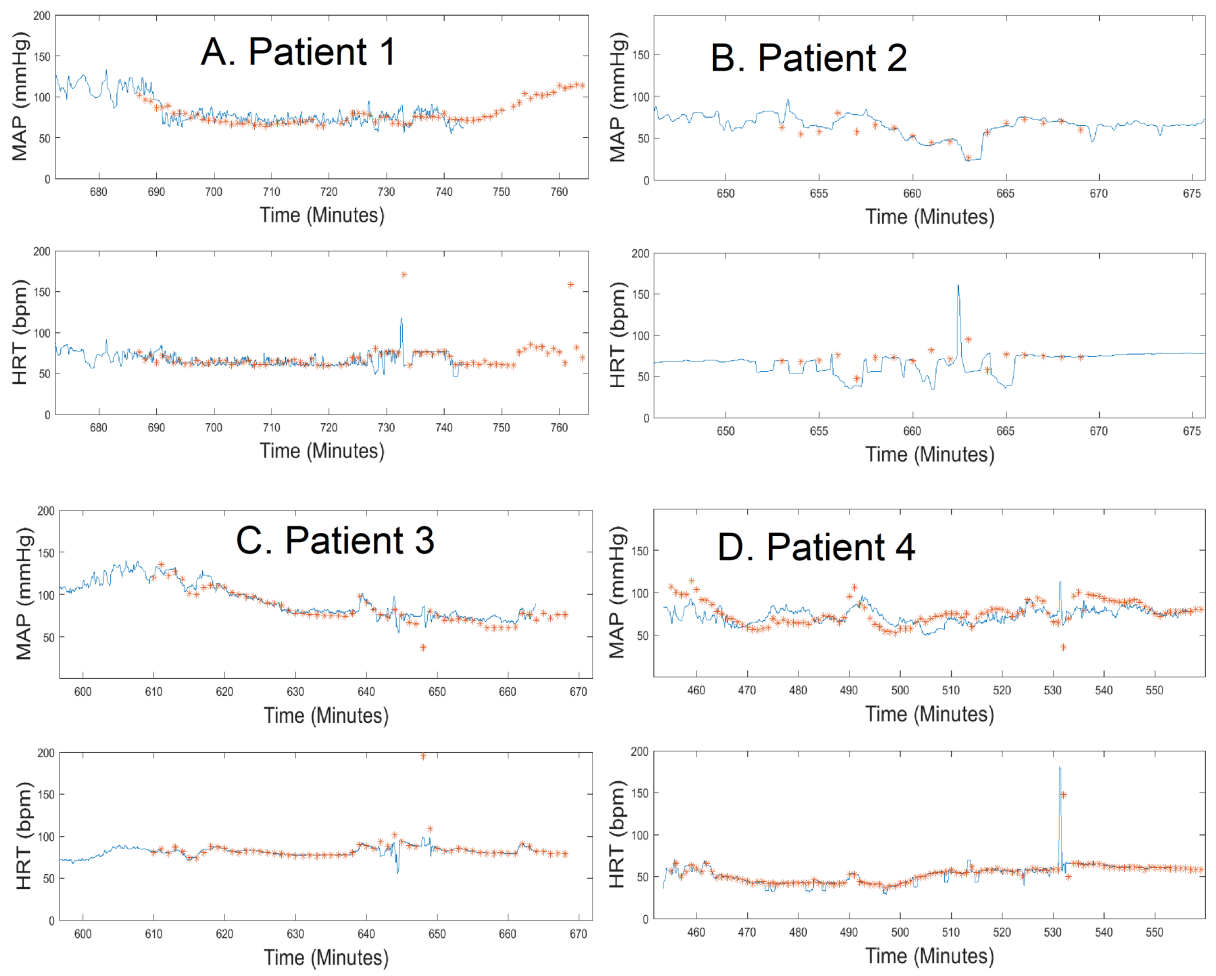
Patient heart rate (HRT) and mean arterial pressure (MAP) comparisons (VitalStream in blue, arterial line in red) for all four patients.

In Figure 1, we present the time domain overlaps of MAP and HRT for all four patients. We compare the data provided by the VS device (blue) with the invasive arterial line (red). We show that there is a good correlation between the data provided by the two different modalities.

**Figure 2:**
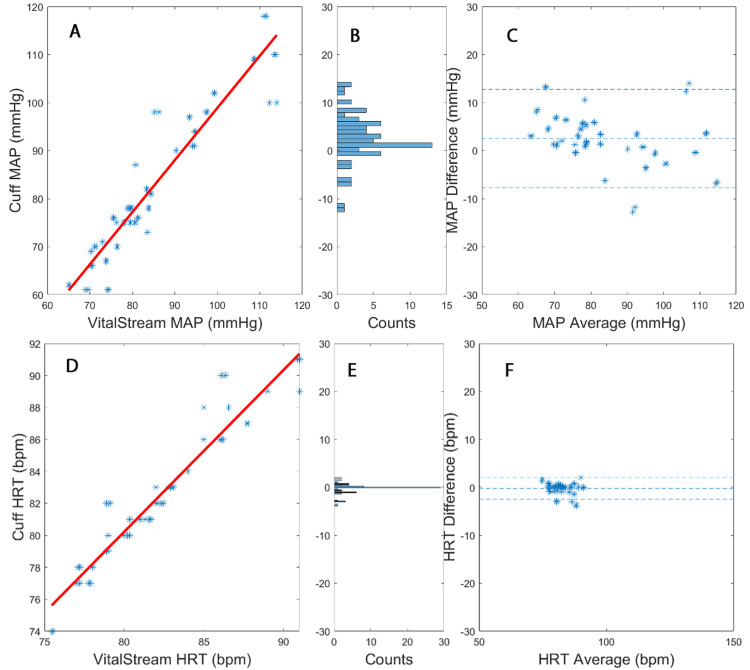
Correlation (A) and Bland-Altman (C) analyses for the mean arterial pressure (MAP) time-domain results presented in Figure 1 for transcatheter aortic valve replacement (TAVR) for patient 2. Panel B presents the count distribution of the Bland-Altman plot. Correlation (D) and Bland-Altman (F) analyses for the heart rate (HRT) time-domain results presented in Figure 1 for TAVR for patient 2. Panel E presents the count distribution of the Bland-Altman plot.

Figure 2 presents the corresponding results of correlation and Bland-Altman analyses for patient 2 undergoing TAVR. The mean difference (standard deviation) is 2.55 mmHg (5.13 mmHg), with a correlation coefficient of 0.94. The lower and upper limits of influence (LOIs) are -7.76 and 12.78, indicating a percent error of 12.0%. For patient 2, the mean difference (standard deviation/SD) is -0.24 bpm (1.13 bpm), with a correlation coefficient of 0.96. The LOIs are -2.51 and 2.01, resulting in a percent error of 2.7%.

**Figure 3:**
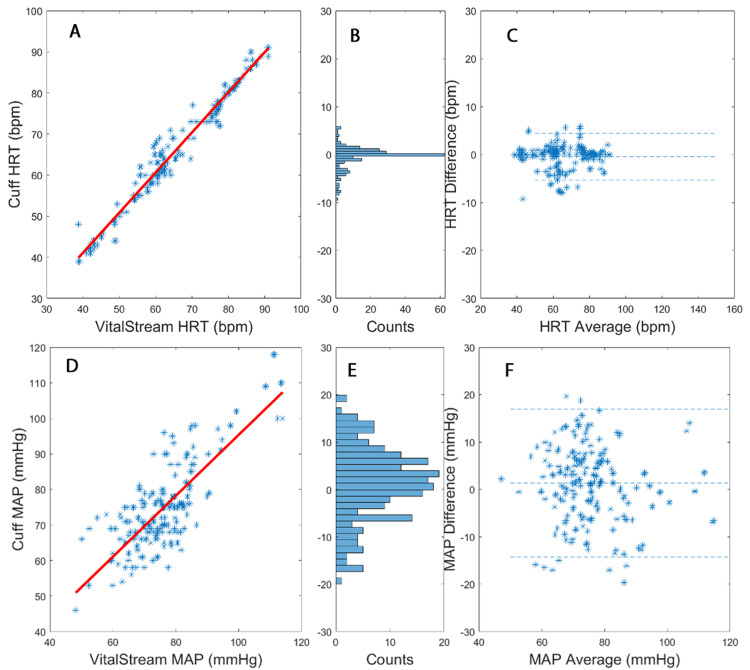
Correlation (A) and Bland-Altman (C) analyses for heart rate (HRT) for all four transcatheter aortic valve replacement (TAVR) patients. Panel B presents the count distribution of the Bland-Altman plot. Correlation (D) and Bland-Altman (F) analyses for mean arterial pressure (MAP) for all four TAVR patients. Panel E presents the count distribution of the Bland-Altman plot.

**Figure 4:**
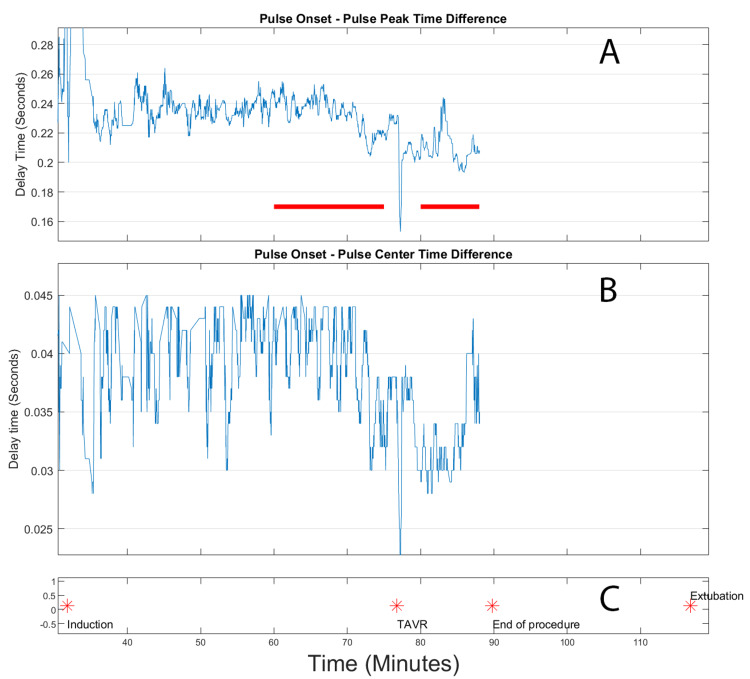
Extracted risetimes for transcatheter aortic valve replacement (TAVR) for patient 1. Top panel (A): Front rise time, in seconds, of the arterial peak. Indicated in this panel by the red lines are the pre- TAVR and post-TAVR deployment time windows used for calculating differences. Center panel (B): Rise time, in seconds, from the onset of the peak to the highest slope point of the front of the pulse. Bottom panel (C): event log, where the stars indicate the time location of the labelled events.

Figure 3 shows the overall correlation and Bland-Altman results for all four patients undergoing TAVR. Figure 4 illustrates a strong correlation between the cuff and VS measurements for HRT. Furthermore, there is a good correlation for MAP. The mean difference (SD) for HRT is -0.45 bpm (2.44 bpm), with a correlation coefficient of 0.98. The lower LOI is [-5.3, 4.4], indicating a percent error of 7.1%. For MAP, the mean difference (with SD) is 1.33 mmHg (7.78 mmHg), with a correlation coefficient of 0.78. The LOI is [-14.2, 16.9], resulting in a percent error of 19.9%.

Risetime findings

Analysis of the first TAVR case (Figure 4) revealed several notable findings. After valve deployment, there was a distinct reduction in both the full and partial risetime measurements. The timing of this improvement showed a strong correlation with the moment of valve deployment, suggesting a direct relationship between the intervention and the observed hemodynamic changes. Throughout the procedure, the signal quality remained excellent with minimal interference, allowing for reliable data collection and analysis.

**Figure 5:**
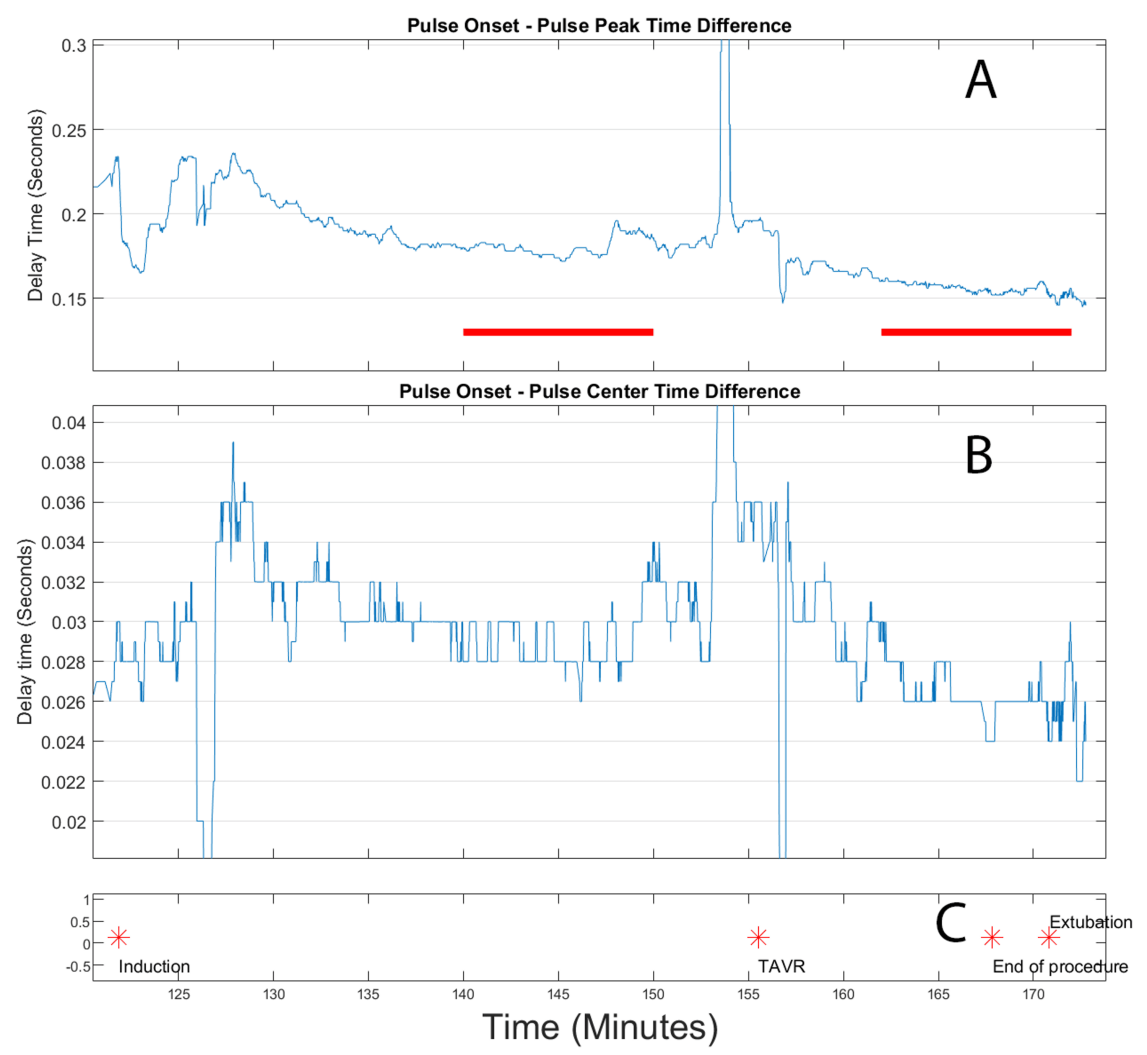
Extracted risetimes for transcatheter aortic valve replacement (TAVR) for patient 2. Top panel (A): Front rise time, in seconds, of the arterial peak. Indicated in this panel by the red lines are the pre-TAVR and post-TAVR deployment time windows used for calculating differences. Center panel (B): Rise time, in seconds, from the onset of the peak to the highest slope point of the front of the pulse. Bottom panel (C): event log, where the stars indicate the time location of the labelled events.

The second TAVR case (Figure 5) revealed a significant improvement in risetime measurements, which occurred simultaneously with valve deployment. This enhancement was particularly evident in the partial front risetime measurement, which showed particularly pronounced changes. Before valve deployment, the baseline measurements remained stable, serving as a reliable reference point for evaluating the post-deployment changes.

**Figure 6:**
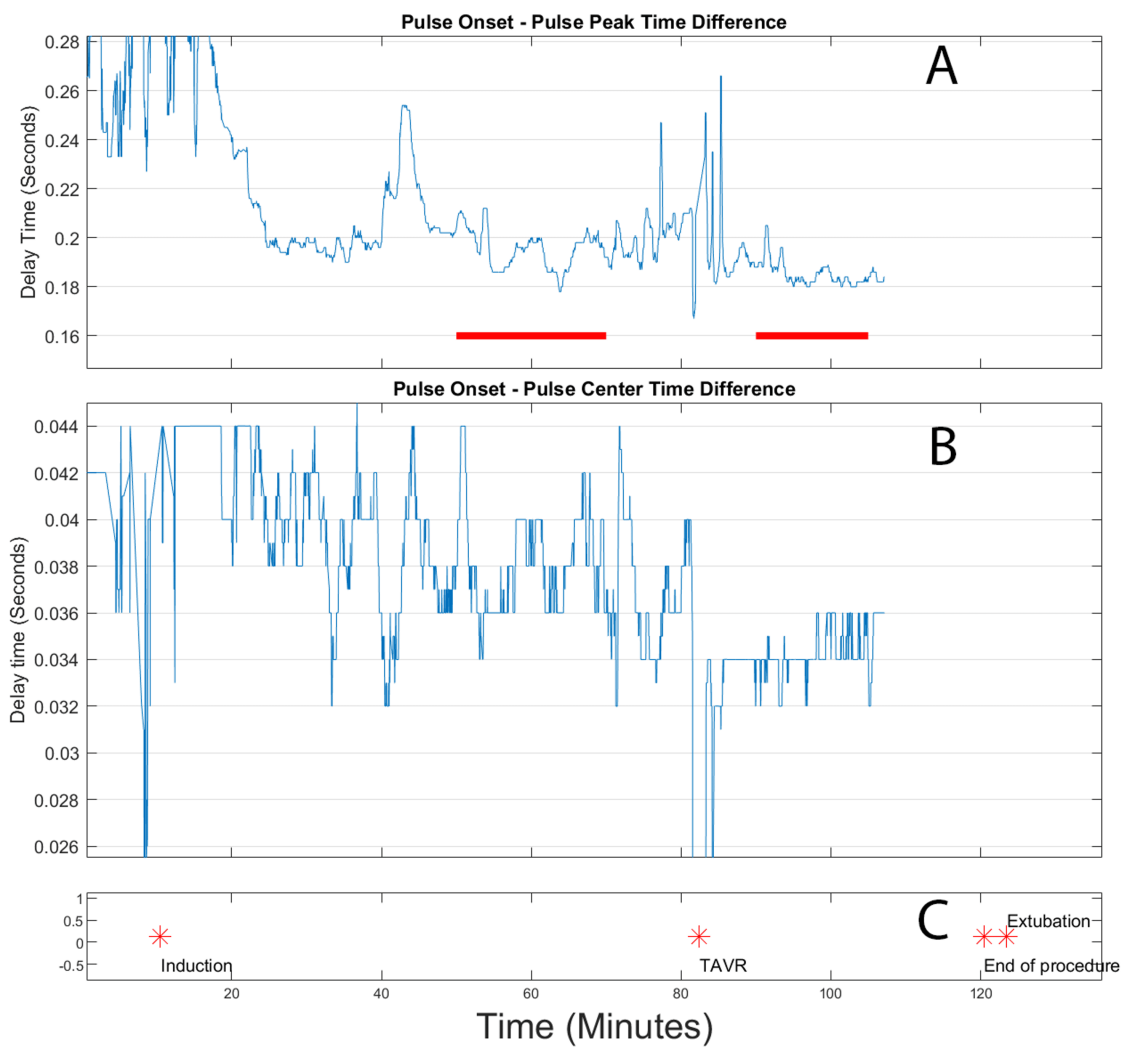
Extracted risetimes for transcatheter aortic valve replacement (TAVR) for patient 3. Top panel (A): Front rise time, in seconds, of the arterial peak. Indicated in this panel by the red lines are the pre-TAVR and post-TAVR deployment time windows used for calculating differences. Center panel (B): Rise time, in seconds, from the onset of the peak to the highest slope point of the front of the pulse. Bottom panel (C): event log, where the stars indicate the time location of the labelled events.

The third TAVR case (Figure 6) further reinforced the patterns observed in the previous cases, exhibiting the same characteristic reduction in risetimes following valve deployment. The temporal correlation between the valve deployment event and the subsequent hemodynamic improvement was evident, providing additional evidence for the consistent nature of this phenomenon. Throughout the entire procedure, the signal maintained high quality, ensuring reliable data collection and enhancing the validity of the observations.

**Figure 7:**
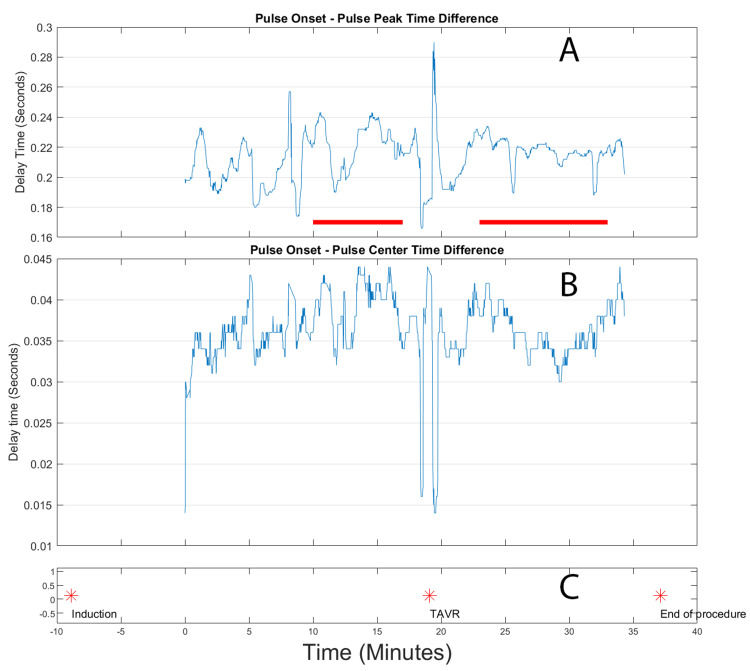
Extracted risetimes for transcatheter aortic valve replacement (TAVR) for patient 4. Top panel (A): Front rise time, in seconds, of the arterial peak. Indicated in this panel by the red lines are the pre-TAVR and post-TAVR deployment time windows used for calculating differences. Center panel (B): Rise time, in seconds, from the onset of the peak to the highest slope point of the front of the pulse. Bottom panel (C): event log, where the stars indicate the time location of the labelled events.

In the fourth TAVR case (Figure 7), monitoring was restricted to the deployment period, thereby narrowing the observation window. Nevertheless, even in the presence of signal noise, the data still exhibited a reduction in partial front risetime measurements. Despite these technical limitations, the case reaffirmed the pattern of improvement observed in the preceding cases, thereby bolstering the consistency of the hemodynamic alterations following valve deployment.

Clinical outcomes

The clinical outcomes for the four patients in this case series are shown in Table 2. As can be seen, there were relatively few significant adverse events noted in the four patients in this series, with only one in-hospital stroke.

**Table 2:** Clinical outcomes ICU: Intensive care unit; LOS: Length of stay; MI: Myocardial infarction.

Outcome	Patient 1	Patient 2	Patient 3	Patient 4
ICU LOS (days)	1	2	0	0
Hospital LOS (days)	1	7	1	1
In-Hospital Death	No	No	No	No
Acute Kidney Injury	No	No	No	No
Pacemaker (Complete Heart Block)	No	No	No	No
In-Hospital Stroke	No	Yes	No	No
In-Hospital MI	No	No	No	No
30-Day Stroke	No	No	No	No
30-Day MI	No	No	No	No
30-Day Death	No	No	No	No
6-Month Stroke	No	No	No	No
6-Month MI	No	No	No	No
6-Month Death	No	No	No	No

## Discussion

The consistent pattern of abbreviated arterial pressure risetimes post-TAVR deployment suggests enhanced hemodynamic performance. This temporal immediacy makes risetime alterations a potential real-time surrogate marker for procedural success, providing immediate feedback on deployment efficacy [16]. Quantitative measurements offer an objective metric for assessing hemodynamic improvement, surpassing traditional qualitative assessments [17]. Notably, partial front risetime measurements showed superior stability, suggesting enhanced reliability for future research.

VS presents a novel non-invasive technology for accurately measuring hemodynamics due to its passive, high-fidelity mode of acquiring arterial pulse signals. To achieve optimal signal fidelity, VS establishes a constant coupling pressure in the finger cuff to partially unload the digital arterial walls, creating a passive listening mode. This contrasts with other systems such as Edward Lifesciences’ Clearsight, which uses the Penaz principle to continuously adjust the finger cuff pressure to zero out the artery’s pulsating pressure [18]. While the pump compromises patient comfort, arterial pulse characteristics are primarily affected by the pump, not physiology. This was determined by a study comparing pulse transit times (PTTs) during exercise measured with the Caretaker, the VS’s predecessor, and the Finapres system [19]. PTTs measured with Finapres were less sensitive to exercise changes and featured an unexplained offset.

Seamless digital journey

The VS platform shows how to create a complete digital ecosystem for the entire perioperative period. This integrated approach changes how complex cardiac procedures are managed and could be a model for various cardiac interventions, such as coronary artery bypass grafting (CABG), atrial fibrillation management, and valvular procedures. The platform’s ability to integrate and analyze data throughout the patient’s journey optimizes perioperative care.

Innovations in patient management

Our findings have revolutionized perioperative monitoring strategies, particularly regarding Swan-Ganz catheterization protocols. Reducing invasive monitoring for CABG patients with preserved ejection fractions, especially in robotic procedures, is driven by robust data from non-invasive VS measurements. While Swan-Ganz catheters remain crucial for valve surgery, their selective application reflects a more nuanced approach. VS’s ability to generate distinct pulse degradation waveforms for atrial fibrillation offers promising non-invasive diagnostic strategies. Our ongoing work aims to identify digital signatures for various valvular conditions, such as aortic stenosis and mitral valve pathologies, both pre-intervention and post-intervention. These digital signatures could revolutionize the longitudinal management of diverse cardiac conditions, providing a unified patient care framework across multiple cardiac pathologies.

Future work

The VS device’s pulse degradation waveform offers a non-invasive, accurate digital signature for AF patients. We aim to identify digital signatures for aortic stenosis and mitral valve conditions, both pre-intervention and post-intervention. This approach could manage patients before, during, and after surgery for conditions like CABG, AF, heart failure, and valve surgery. Future research includes larger validation studies, correlating results with long-term outcomes, developing automated real-time monitoring systems, and investigating risetime patterns in different valve types.

Limitations

This case series has some limitations. The sample size is small (four cases), and signal quality varies across cases, affecting data consistency. Also, limited baseline data affected the comparison of pre-procedure and post-procedure measurements.

This case series suggests that the VS system advances perioperative cardiac monitoring, potentially improving clinical outcomes and procedural efficiency. Reduced invasive monitoring and enhanced real-time feedback warrant further investigation in larger, multi-center studies.

## Conclusions

This analysis demonstrates that TAVR deployment results in immediate improvements in arterial pressure wave characteristics, particularly showing decreased risetimes. These changes were consistent across cases and temporally correlated with valve deployment, even in the presence of varying degrees of signal quality. Notably, partial front risetime measurements exhibited greater stability and less interference, highlighting their potential reliability. These findings suggest that risetime analysis could serve as a valuable tool for real-time procedural monitoring and outcome assessment during TAVR.
